# Innovative approaches to genome editing in avian species

**DOI:** 10.1186/s40104-018-0231-7

**Published:** 2018-02-09

**Authors:** Caitlin A. Cooper, Timothy J. Doran, Arjun Challagulla, Mark L. V. Tizard, Kristie A. Jenkins

**Affiliations:** CSIRO Health and Biosecurity, Australian Animal Health Laboratory, Private Bag 24, Geelong, VIC 3220 Australia

**Keywords:** Avian, CRISPR, Genome engineering, PGCs, Sperm, TALEN

## Abstract

The tools available for genome engineering have significantly improved over the last 5 years, allowing scientist to make precise edits to the genome. Along with the development of these new genome editing tools has come advancements in technologies used to deliver them. In mammals genome engineering tools are typically delivered into in vitro fertilized single cell embryos which are subsequently cultured and then implanted into a recipient animal. In avian species this is not possible, so other methods have been developed for genome engineering in birds. The most common involves in vitro culturing of primordial germ cells (PGCs), which are cells that migrate through the embryonic circulatory system to the developing gonad and colonize the gonad, eventually differentiating into the gonadocytes which produce either sperm or ova. While in culture the PGCs can be modified to carry novel transgenes or gene edits, the population can be screened and enriched, and then transferred into a recipient embryo. The largest drawback of PGC culture is that culture methods do not transfer well across avian species, thus there are reliable culture methods for only a few species including the chicken. Two newer technologies that appear to be more easily adapted in a wider range of avian species are direct injection and sperm transfection assisted gene editing (STAGE). The direct injection method involves injecting genome engineering tools into the circulatory system of the developing embryo just prior to the developmental time point when the PGCs are migrating to the gonads. The genome engineering tools are complexed with transfection reagents, allowing for in vivo transfection of the PGCs. STAGE utilizes sperm transfection to deliver genome engineering tools directly to the newly fertilized embryo. Preliminary evidence indicates that both methodologies have the potential to be adapted for use in birds species other than the chicken, however further work is needed in this area.

## Background

The application of precision genome engineering (PGE) in animal agriculture has great potential, with many in the field predicting that this technology will transform livestock breeding. Among other things PGE tools enable the rapid introduction of beneficial naturally occurring mutations that already exist within a species or closely related species into elite breeding animals, a process known as precision breeding. Since PGE tools are so specific they can be used to introduce beneficial alleles, however unlike traditional breeding there is no risk of also introducing deleterious or unwanted traits that are chromosomally linked to the beneficial allele. While applying these PGE tools in avian species presents additional technical challenges compared to many mammalian species, we now have the technology to create precise, targeted modifications to the chicken genome. Genome editing tools have recently been applied in the chicken with both TALEN and CRISPR-Cas9 used to create targeted gene knockout chickens. The impacts of this technology could lead to improved efficiency and sustainability of poultry production to help meet the challenges associated with global food security. Specific innovations that result from gene editing technology will lead to new approaches in many areas including managing disease, improving welfare, increasing food safety and enhancing the production and safety of vaccines that are grown in chicken eggs. It is possible that the latest developments in gene editing technology may help to reduce or remove the two major barriers to the acceptance and application of genetic engineering technology in animal agriculture; regulatory approval and public perception.

Multiple labs rely on primordial germ cell (PGC) culture to develop genome edited chickens. Avian embryonic PGCs migrate through the vasculature on their path to the gonad where they become the sperm or ova producing cells. This unique feature of avian PGC migration through blood has led to a transformational advance in the generation of genetically engineered chickens. This involves establishing PGC cultures in vitro, introducing genetic modifications into cultured cells, expanding the modified cells into clonal populations and injecting selected cells into recipient embryos to create gonadal chimeras. The chimeras are then bred to create germline edited offspring. This works well for chickens where PGC culture methods have been well established. Unfortunately PGC culturing is not straightforward for other avian species, including poultry species closely related to the chicken. Our lab has been working on two methods to apply genome engineering tools (transgenesis and editing) in a wider range of avians – direct injection and sperm transfection assisted gene editing (STAGE). This review provides an overview of how these technologies have been developed and the possibilities to them apply in: developmental biology in species such as quail and zebra finch which are both excellent model organisms; agriculture in species such as turkeys and ducks to help improve production traits, improved welfare and safer food products; and finally in conservation for the genetic rescue of the many endangered bird species around the globe.

## Precision genome engineering tools and their use in chickens via a PGC culture approach

The use of PGE tools was first described almost 2 decades ago with the demonstration of targeted integration of exogenous DNA at double stranded breaks (DSBs) induced by rare-cutting endonucleases in eukaryotic cells [1]. Since this original paper 3 major classes of PGE tools have been described. They are zinc finger nucleases (ZFNs), transcription activator-like effector nucleases (TALENs), and clustered regularly interspersed short palindromic repeats (CRISPR), which are all used to introduce DSBs and allow targeted genome editing.

ZFNs where seen as a significant step forward in the PGE field as they allowed the assembly of customised DNA binding proteins. Typically a ZFN is made up of three or four zinc fingers (ZFs) fused to a non-specific nuclease *Fok*I [2, 3]. The ZFs, which are transcription factors, consists of 30 amino acids that recognise trinucleotides. ZF library’s that are specific to all 64 possible trinucleotides combinations are available allowing the design of functional ZFN to target virtually any sequence of interest. Although in theory it is possible to assemble ZFNs to any sequence in the genome there can be complications with their assembly and some ZFs have been shown to influence the binding integrity of other adjacent ZFs leading to inefficient binding [4, 5]. In 2002, the first successful germline transmission of a ZFN induced mutation was reported in *Drosophila melanogaster* [6]. Since then, several reports have been published the use of ZFNs for targeted genome engineering for diverse range of applications in genetic modification and gene therapy applications [7]. However, ZFN mediated gene editing in poultry is yet to be reported.

In 2011 an alternative site specific nuclease for use in eukaryotic cells, TALENs was described [8]. TALENs were developed from TALE DNA binding motifs from the proteins derived from the bacterial plant pathogen *Xanthomonas campestris* [9]. Similar to ZFNs, TALENs utilise a *Fok*I domain which in this case is coupled with TALEs which are made up of 33–35 amino acid blocks in tandem repeat that are able to recognise a single nucleotide. In comparison to ZFNs, TALENs are easier to construct and their smaller size results in less steric hindrance. Their ability to be multiplexed has also been demonstrated, making them a more desirable gene editing tool [8].

The application of TALENs to induce gene editing has been demonstrated in a range of animal species including chickens. TALENs have been used to generate ovalbumin (OVA) knock out (KO) chickens [10]. In this study cultured PGCs were transfected with plasmids encoding OVA-TALENs [10]. This resulted in 33% of the PGCs cultures containing deletions in the OVA gene that ranged between 6 and 29 nt. The PGCs containing the OVA modifications were transplanted into recipient embryos and the chimeric roosters were raised to sexual maturity. These chimeric roosters generated OVA heterozygous knockout chicks with an efficiency of 10% [10].

More recently, Taylor et al. used TALEN in combination with homology directed repair (HDR) to produce sterile hens [11]. Similar to Park et al., cultured PGCs were transfected with plasmids encoding TALENs, in this case targeting the DDX4 (vasa) locus. In this study a HDR template containing a reporter (GFP-2A fused to puromycin) was also included to allow selection of the targeted PGCs. After two weeks of culture 8.1% of PGCs were found to be expressing GFP indicating successful HDR [12]. Male cells which were heterozygotes for the modifications were then transplanted into recipient embryos and raised to sexual maturity. One of the founder roosters was mated, resulting in modified offspring at an efficiency of 6% [12].

The next major advancement in the field was in 2013 when CRISPR, which is a part of the microbial adaptive immune system, was adapted for genome editing in eukaryotic cells [13]. In bacteria and archaea the CRISPR locus acquires foreign DNA from invading viruses and plasmids and insert it into spacers, before transcribing them into CRISPR RNAs (crRNAs) to guide the ribonucleoprotein complex to recognise and cleave invading nucleic acids [14]. Before it was adapted for use in eukaryotic cells it was demonstrated that by changing the seed sequence in the crRNA, Cas9 could be programmed to introduce site specific DSBs in target DNA adjacent to GG containing protospacer-adjacent motifs (PAM) [15]. It was then shown that crRNA and tracrRNA structures can be fused to generate a single guide RNA (sgRNA) for Cas9-mediated targeted genomic DSBs [13, 16].

In short, Cas9-mediated genome editing in eukaryotic cells requires expression of Cas9 protein with a nuclear localization signal and a sgRNA sequence with appropriate promoters in a cloning vector. The only constraint in the design of an active guide RNA is the requirement of a PAM sequence of 5′-NGG-3′ to be located adjacent to the target sequence in the genome.

CRISPR is widely considered the most user friendly PGE tool as unlike ZFNs and TALENs it relies on a single protein to mediate the DSBs resulting in less steric hindrance problems and allows the target sequence to be easily changed by using a different guide RNA sequences [17]. Another advantage is the ability to simultaneously target multiple genes at once by expressing multiple sgRNAs [13].

Many groups have explored the use of CRISPR/Cas9 for genome editing in a variety of species including chickens. The first study using CRISPR in chicken was published in 2015 and involved the electroporation of chicken embryos with plasmids encoding Cas9 and guide RNAs against the transcription factor PAX7 [18]. This study demonstrated that targeting vectors into the neural tube and dorsal dermomyotome of E3.5 embryos resulted in a reduction of PAX7 expression between 80%–90% compared to the control embryos. These results clearly demonstrated that CRISPR was able to efficiently mediate gene editing in chicken embryos and concluded that it will be a valuable tool to study the molecular mechanisms regulating development in the chicken [18].

Two reports of germline gene editing in the chicken were published in April 2016 [11, 19]. Dimitrov et al. used a combination of CRISPR and HDR to target the chicken immunoglobulin heavy chain locus in cultured PGCs [19]. Cultured PGCs were electroporated with two plasmids, one encoding a sgRNA and Cas9 and the second encoding a HDR template. PGCs containing the desired modifications were enriched for using antibiotic resistance. The modified PGCs were then injected into recipient embryos to generate chimeric birds that were raised to sexual maturity and progeny assessed for the modification. In this study they found germline transmission rates ranging from 0 to 96% from their 13 chimeric roosters. This study demonstrated the first successful use of CRISPR/Cas9 assisted HDR of donor DNA in the chicken [19].

Oishi et al. generated ovomucoid (OVM) KO birds using cultured PGCs and CRISPR [11]. The cultured PGCs were transfect with a plasmid encoding Cas9 and a guide RNA against OVM. In this case no HDR template was used so NHEJ was relied on to generate the mutations. Using this approach they found deletions ranging from 1 to 21 bp in OVM. Interestedly in the 13 clones sequenced no insertions were detected [11]. As with the previously described studies the modified PGCs were enriched for prior to injection into recipient embryos. In this study the cultured PGCs were generated from a different line of birds to the recipients which allowed for colour selection of donor derived chicks from the chimeric roosters. Of the donor derived chicks (average of 73%), 53% were found to contain mutations in OVM. In this study they also went on to produce homozygous OVM KO birds which were healthy but they did not examine if the KO birds were able to produce viable eggs or reproduce [11].

## Non PGC culture approaches for gene editing in avians

It is clear that the most recent breakthroughs in the generation of genetically engineered birds have come from the use of PGC culture. PGC cultures have been used for generating gene KOs using gene targeting [20], TALENS [10, 12] and CRISPR [11, 19]. However for many agricultural and model avian species and lines, PGC cultures are not available and alternate approaches are required.

In 2013, Tyack et al. reported a new method for producing transgenic chickens via direct in vivo transfection of PGCs. In this study they used the miniTol transposon system made up of two plasmids; the first plasmid contained the EGFP transgene under the control of the CAGGS promoter and flanked by the Tol2 ITRs (pMiniTol-EGFP); and the second plasmid (pTrans) encoded the Tol2 transposase under the control of the CMV immediate-early promoter for in trans expression of the transposase and subsequent transposition of miniTol-EGFP from plasmid into the genome of the transfected cells [21]. In this study the two plasmids were combined and formulated with Lipofectamine® 2000 before being intravenously injected into stage 14 HH embryos (approximately d 2.5 of embryogenesis). Using this approach they were able to generate chimeric roosters which were capable of passing the transgene onto the next generation [21]. To date only the production of transgenic chickens has been published using this method, however we believe that plasmids encoding gene editing tools such as TALENS and CRISPR could be delivered via this direct injection approach to produce gene edited birds.

In the published work with PGC cultures, plasmids encoding gene editing machinery were transfected into the cells [10–12, 19]. Park et al. transfected the PGCs with a combination of three plasmids, two TALEN encoding plasmids which targeted OVA and a CMV GFP plasmid [10]. The expression of GFP allowed the transfected cells to be enriched for using fluorescence-activated cell sorting one day post transfection. These enriched cells were then injected into recipient embryos [10].

Oishi et al. transfected their cultured PGCs with a single plasmid, that encoded human CAS9, a sgRNA targeting OVM and a gene encoding antibiotic resistance [11]. They then used transient antibiotic selection to enrich for the modified PGCs before injecting them back into recipient birds [11].

It is feasible that for both of these studies the transfection of the PGCs could have been carried out in vivo instead of in culture. We believe that as demonstrated with the miniTol2 transposon plasmids that PGCs transfected in vivo with PGE plasmids would result in edited PGCs that would migrate to the germinal ridge to produce gonadal chimeric birds. The gonadal chimeric roosters could be identified at sexual maturity by assays on their semen. The roosters identified to have the highest percentage of edited semen could then be mated with wildtype females to produce G1 offspring that are heterozygous for the edit or deletion of interest. The G1 offspring could then be mated to produce homozygous edited or knock out birds.

Using the direct in vivo transfection approach we believe it should be possible to obtain CRISPR induced NHEJ by injecting a single plasmid encoding Cas9 and a sgRNA against the gene of interest complexed with lipofectamine intravenously into stage 14 HH embryos. It should also be possible to delete a region of a gene by using a single plasmid containing two sgRNAs against the gene of interest. It is possible to deliver the two guide RNAs in separate plasmids but in vitro work we have performed has demonstrated that the use of a single plasmid containing both sgRNAs is more efficient at generating the desired deletion.

Using PGC culture, targeted edits using homology directed repair (HDR) have been demonstrated using both TALENs and CRISPR [12, 19]. In Taylor et al. cultured PGCs were transfected with plasmids for the TALEN pair along with a plasmid encoding the reporter cassette flanked with homology arms to allow HDR [12]. While in Dimitrov et al. a plasmid encoding Cas9 and a guide RNA was electroporated along with a HDR plasmid encoding a loxP site and antibiotic selection cassette flanked with homology arms [19]. We believe that the knockout and reporter integration achieved in these papers may also be possible using the direct injection approach. The best approach to provide the HDR template for the direct injection would need to be optimised but may include the transfection of a plasmid or DNA oligo providing the template for HDR being alongside the plasmid expressing the CRISPR and sgRNAs.

A downfall of the direct in vivo transfect approach is the inability to enrich for a modified PGC population as was done in the studies using PGC culture. This may result in a lower frequency of modified G1 offspring from the gonadal chimeric roosters being generated using this approach. Although this may be a disadvantage for lines of birds for which PGC cultures are available there are a number of species and lines of avians for which PGC cultures are unavailable. In addition to the chicken the direct injection method has been use to successfully transfect quail PGCs in vivo with piggyBacCMV-GFP. These PGCs remigrated to the gonad and successfully colonised it, with clear GFP expression seen on embryonic d 12 [22]. These results further suggest that for avian species without PGC cultures the direct in vivo transfection approach is one of a limited number of methods available with the potential to generate edited birds.

Another germ cell culture free approach that could be used is sperm transfection assisted gene editing (STAGE) [23]. This method involves transfecting sperm with *Cas9* mRNA and guide RNA and then using the transfected sperm for artificial insemination in hens. STAGE was designed to harness the ability of sperm to deliver nucleic acids and combine that with recently developed gene editing systems such as CRISPR/Cas9. In the past researchers have attempted to use sperm as a delivery mechanism for transgene constructs [24]. While the sperm proved very effective at delivering DNA constructs, transgene integration into the genome remained a huge hurdle [25]. This research did lay the foundation to show that sperm cell transfection is possible, and that transfected sperm are both viable and capable for fertilization.

STAGE is particularly relevant to avian species as the current methods that could be used to produce gene edited birds take two generations. Editing PGCs in culture then transferring them to developing embryos [11] and direct in vivo transfection of circulating PGCs in embryos [21] both result in gonadal mosaic birds. These birds must be raised to sexual maturity and then mated to generate a bird containing the desired edit in all of its cells, with transmission rates ranging from 0.5% to 40%. While setting up a large scale breeding program is common practice with domesticated species like the chicken, it may present an obstacle for researchers looking to explore gene editing in non-domestic bird species.

STAGE is designed to cause mutations in the early zygote, preferably occurring in the single cell zygote to generate full knockout animals in a single generation, however it can also causes gene mutations in the multi cell zygote, leading to mosaicism [23]. STAGE enables editing in the early zygote because it delivers *Cas9* mRNA and synthesized sgRNAs as opposed to plasmids containing these components. The STAGE method utilizes RNA based components because avian oocytes and early embryos, like oocytes and early embryos from most species, are transcriptionally quiescent [26]. Early zygotic development is directed by maternal RNA deposited in the cytoplasm prior to ovulation, with the embryo eventually becoming transcriptionally active. This process is known as the maternal to zygotic transition, and in chickens it occurs when the embryo reaches stage X and contains more than 20,000 cells [27]. Recent research has shown that in the chicken the male also contributes RNA to the zygote that helps direct early embryonic development [28]. How RNA is packaged and stored in chicken sperm is unknown, however understanding and being able to mimic this process may improve the efficiency of STAGE.

The majority of the gene mutations generated when using STAGE are different from those typically observed when delivering CRISPR/Cas9 components to cells in culture or to mammalian oocytes or fertilized zygotes. While most CRISPR/Cas9 induced mutations are clustered within ten to fifteen base pairs of the PAM site, the mutations induced by STAGE often occur fifty to two hundred base pairs away from the PAM site [23]. It is unclear why this is occurring, which is further compounded by the lack of knowledge about the DNA repair mechanisms present in the early chicken zygote. It is possible that these mutations occur as a result of errors taking place during DNA break repair, but additional studies investigating DNA repair in avian zygotes are needed to further elucidate the mechanisms underpinning the mutations resulting from STAGE.

**Fig. 1:**
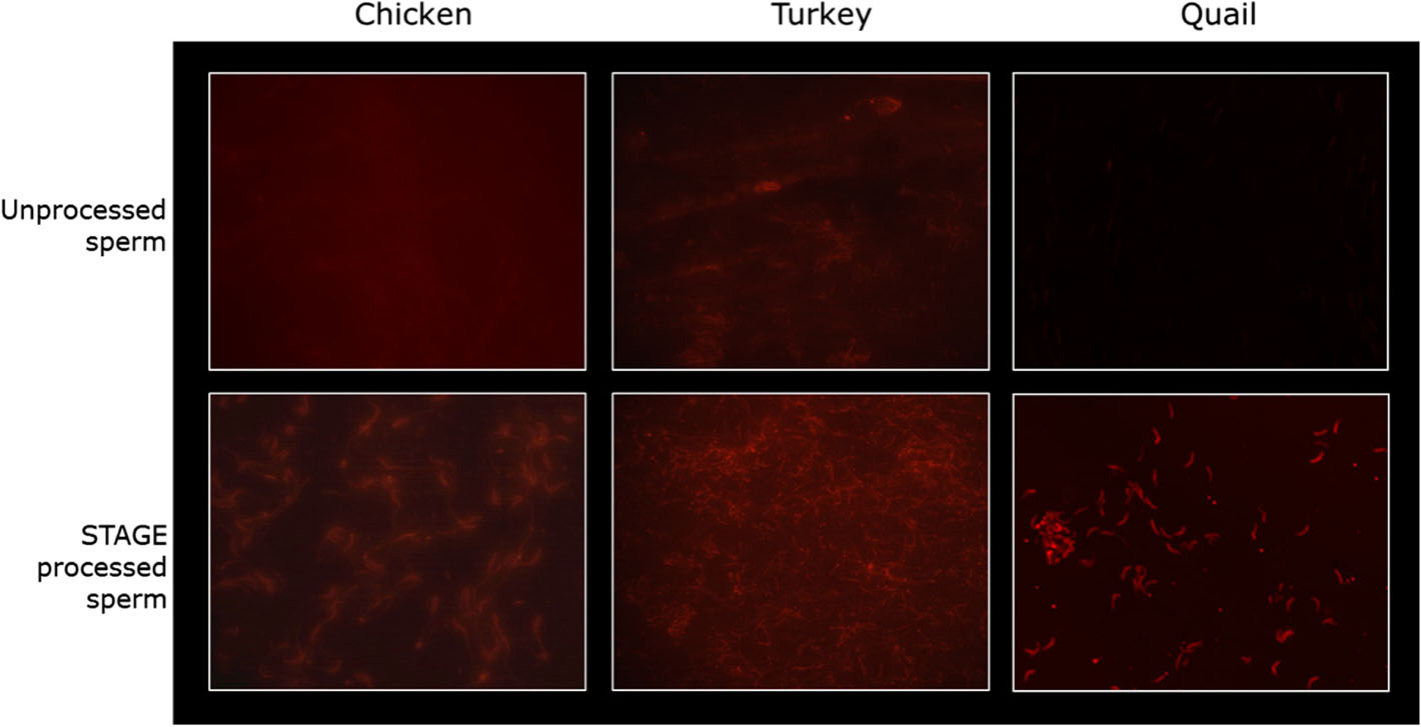
Chicken, turkey, and quail sperm incubated with Lipofectamine® 2000 and BLOCK-iT™ fluorescently labelled RNA. The top panel shows unprocessed sperm, where poor transfection of the labelled RNA to the sperm is seen. The bottom panel shows STAGE processed sperm, where these is clearly increased transfection of the labelled RNA to the sperm. Quail pictures taken by Olivier Serralbo of the Monash Transgenic Quail Facility

Due to the straight forward nature of the protocol it is likely that STAGE could be effective for generating gene knockouts in other avian species besides the chicken. Given that STAGE involves minimally invasive procedures and only very basic laboratory equipment it has the potential to be used by a variety of scientists with a wide range of species. Preliminary results indicate that using the STAGE protocol for sperm preparation with quail, chickens, and turkeys leads to successful transfection of RNA. Sperm was washed and then incubated with Lipofectamine® 2000 and a fluorescently labeled RNA (BLOCK-iT™, Thermo Fisher). The results indicate that sperm from all three species remained motile during the transfection process and that the RNA was effectively delivered to the sperm (Fig. 1). Based on these results it appears that the STAGE protocol can deliver RNA to the sperm of multiple bird species, however quail, chickens, and turkeys are all Galliformes, so more work must be done to determine how applicable these conditions are for a broader range of avian species.

Overall STAGE is a promising new method which has the potential to be applied to many different avian species including quails and turkeys. However STAGE generates a high proportion of single base mutations between 50 and 200 base pairs distal to the PAM site, making it still effective for gene knockouts, but less effective for precise editing. To better understand the mechanism underlying these mutations further research is needed to elucidate the DNA repair mechanisms present in the early chicken zygote. In addition as a recent protocol there is significant scope for improving and optimizing the STAGE method to improve its use in gene editing.

## Conclusion

Along with the development of PGE tools improvements in the technologies that enable access to the germline to generate modified lines of birds have also occurred. These include advances such as more refined protocols for PGC culture and the delivery of PGE tools to PGCs, direct injection of genome editing tools into the bloodstream of early embryo [21], and the transfection of PGE tools directly into sperm [23]. It is reasonable to foresee that these techniques could be applied to any avian species that captive breeding is possible and a reasonable amount of genome sequence data is available. Obtaining modified avian species through the culture and in vitro modification of PGCs has advantages, including that a recipient embryo can be treated to ablate resident PGCs, improving gonadal colonization rates of the modified donor PGCs, thus leading to a higher rate of fully modified offspring. The only constraint of this approach is the need for species specific development of PGC culture conditions as prior work has shown that PGC culture conditions can vary greatly between species. Direct injection has the advantage of shortening the time frame for producing a modified chicken as there is no retrieval, culture, in vitro modification, or selection of PGCs since the modifications made to the PCGs occur in situ. Sperm transfection assisted gene editing (STAGE) reduces timeframes even further as the first generation of animals will carry the modification [23], however it is still in the early stages of development and it is not yet clear if it is as flexible, efficient or robust as PGC culture or direct injection. Together all three methods make generating targeted modifications in a wide variety of avian species possible.

For the poultry industry, in relation to chicken meat or egg production for food, there are many opportunities to apply gene editing. This includes the ability to remove deleterious homozygous recessive alleles in genes that are found in close proximity to beneficial alleles for various production traits. These occur from time to time and are difficult to deal with by conventional breeding due to their genetic linkage (i.e. they do not segregate easily) but could be removed or replaced with positive or neutral alleles using gene editing techniques. Maintaining healthy chickens throughout the poultry production chain has a high impact on food safety, production costs, and food availability, thus there are significant opportunities for gene editing based solutions in this space. Disease resistance traits are also an appealing opportunity with the potential to use gene editing to remove cell surface molecules that viruses or bacteria use as binding sites. Additional traits that are candidates for gene editing are the allergens present in egg proteins such as OVM, OVA, ovotransferrin and lysozyme. For most of these proteins the allergenic epitopes are known and editing of the amino acid sequence in these regions could be used to ablate those epitopes to generate lines of poultry which produce “hypoallergenic” eggs [29].

In relation to the poultry industry and food production the most important issue that will determine the value of these new technologies into the future is the status of the birds that are generated – in particular whether birds resulting from gene editing will be classed as genetically modified organisms (GMOs) or not. This issue has been at the center of a review of the National Gene Technology regulations conducted by the Australian Government’s Office of the Gene Technology Regulator [30]. Meanwhile in the US the Coordinated Framework of Government instruments has led to the issue of an industry guidance note 187 [31]. In Europe the regulatory authorities have not made any statements regarding these technologies, however the European Academies Science Advisory Council has made a statement in support of classifying products of gene editing as non-GMOs [32]. Whatever the outcome of this international debate these technologies have completely changed what is possible in the science of studying and manipulating the biology of avian species. While the future of gene editing in avian species has great potential for applications in biomedical research, conservation, and agriculture, most applications to date have been focused on the chicken. The continued development of novel techniques for delivery of PGE tools such as direct injection and STAGE will hopefully open up opportunities for gene editing in a wider number of avian species.

## Abbreviations

CRISPR: Clustered regularly interspersed short palindromic repeats
DSBs: Double stranded breaks
GMOs: Genetically modified organisms
HR: Homologous recombination
KO: Knock out
NHEJ: Non-homologous end joining
OVA: Ovalbumin
OVM: Ovomucoid
PAM: Protospacer adjacent motif
PGC: Primordial Germ Cell
PGE: Precision genome engineering
sgRNA: Single guide RNA
STAGE: Sperm transfection assisted gene editing
TALENs: Transcription activator-like effector nucleases
ZFNs: Zinc finger nucleases
